# Long-Term Prognosis of Hyperferritinemia Induced by Intravenous Iron Therapy in Patients Undergoing Maintenance Hemodialysis: A 10-Year, Single-Center Study

**DOI:** 10.1155/2020/8864400

**Published:** 2020-12-18

**Authors:** Sayako Maeda, Ryo Konishi, Takuya Morinishi, Yoko Shimizu, Haruomi Nishio, Koji Takaori

**Affiliations:** Department of Internal Medicine, Division of Nephrology, Japanese Red Cross Otsu Hospital, 1-1-35 Nagara, Otsu, Shiga 520-8511, Japan

## Abstract

Optimal ferritin level in hemodialysis patients between Japan and other countries is controversial. Long-term side effects of iron supplementation in these patients remain unclear. We aimed to elucidate whether past hyperferritinemia in hemodialysis patients was associated with high risk of death and cerebrovascular and cardiovascular diseases (CCVDs). This small retrospective cohort study included approximately 44 patients unintentionally supplemented with excessive intravenous iron. A significantly higher risk of CCVDs was observed in patients with initial serum ferritin levels ≥1000 ng/mL than in the remaining patients. High ferritin levels slowly decreased to <300 ng/mL in a median of 24.2 (10.5–46.5) months without treatment. However, compared with the remaining patients, only patients whose ferritin levels did not decrease to <300 ng/mL steadily had a significantly higher risk of all-cause death (hazard ratio, 9.6). Long-term hyperferritinemia due to intravenous iron therapy is a risk factor for death in maintenance hemodialysis patients. For a prolonged better prognosis, intravenous iron should be carefully administered so as to avoid hyperferritinemia in patients with hemodialysis.

## 1. Introduction

Iron supplementation with recombinant human erythropoietin (rHuEPO) is crucial in improving renal anemia in patients undergoing hemodialysis. Iron can be provided through the intravenous and oral routes. The intravenous route, which can achieve target hemoglobin levels and reduce the requirement for rHuEPO, is preferred in patients undergoing dialysis [1, 2].

However, an increasing number of studies report that iron overload in hemodialysis patients might promote atherosclerotic plaque instability; increase the risk of ischemic cardiovascular complications, immune dysfunction, and infection; and trigger nonalcoholic fatty liver disease [3–5]. In September 2017, the Pharmacovigilance Committee of the European Medicines Agency considered convergent publications on iatrogenic hemosiderosis in patients undergoing dialysis and requested that companies holding marketing authorization for iron products should investigate the risk of iron overload in patients undergoing dialysis. Conversely, the recently published PIVOTAL study confirmed the efficacy and safety of intravenous iron for the treatment of anemia in hemodialysis patients [4, 6].

The reported levels of serum ferritin, a representative marker of iron stores, in hemodialysis patients, are very different between Japan and Western countries. The amount of intravenous iron administered is higher in Western countries than that in Japan; consequently, the levels of serum ferritin are significantly higher in hemodialysis patients in Western countries than those in Japan [4, 7]. In studies from Japan, the highest ferritin levels that were reported to be associated with increased mortality risk were ≤200 ng/mL [8, 9]. Conversely, in a 2011 study from the USA, the median ferritin level in hemodialysis patients was 650 ng/mL, and 34% of the patients had ferritin values >800 ng/mL [4]. The Japanese Society of Dialysis Therapy (JSDT) guidelines recommend iron administration with a target serum ferritin level <300 ng/mL in hemodialysis patients [10]. These results highlight the large discrepancy in intravenous iron use and target serum ferritin levels between Japan and Western countries.

Possible explanations of these conflicting findings include differences in methods of adverse event adjudication, baseline differences in patient characteristics, and short follow-up periods in the majority of the studies [11].

Until March 2008, at our institution, 56 outpatients undergoing chronic dialysis therapy were treated by some physicians who did not adhere to the Japanese guidelines. Consequently, in about half of these patients, the serum ferritin levels were very high (554–2930 ng/mL) due to the intravenous iron supplementation following hemodialysis initiation.

In this retrospective cohort, we report the long-term prognosis of patients with iatrogenic hyperferritinemia and discuss the controversies regarding the safety of intravenous iron therapy in patients undergoing maintenance hemodialysis.

## 2. Materials and Methods

### 2.1. Study Design

This retrospective cohort study included 56 outpatients undergoing chronic hemodialysis who regularly visited our Japanese Red Cross Otsu Hospital three times a week between April 2008 and March 2018. All patients provided written informed consent, and the protocol for the research project has been approved by the Ethics Committee of Japanese Red Cross Otsu Hospital (approval no. 491, 2018). The study conforms to the provisions of the Declaration of Helsinki (as revised in Fortaleza, Brazil, October 2013).

### 2.2. Participant Selection and Follow-Up

**Figure 1:**
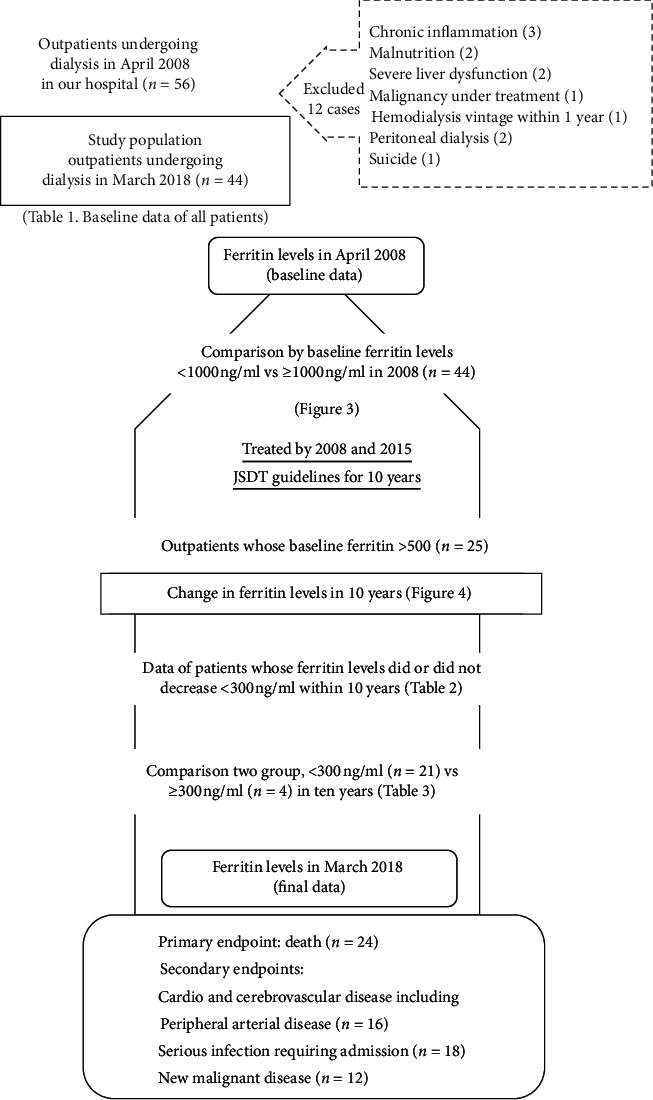
Study flowchart. JSDT, the Japanese Society of Dialysis Therapy.

Figure 1 shows the study flowchart. All the 56 outpatients started dialysis therapy in our hospital. All patients were administered intravenous iron through the blood circuit immediately after introducing the dialysis treatment. We excluded 12 patients both at baseline and at the end of study based on the presence of chronic inflammation (C-reactive protein >0.3 mg/dL, and their ferritin levels: 2930, 2610, and 890 ng/mL), malnutrition (serum albumin <3.3 g/dL: 1640 and 679 ng/mL), severe liver dysfunction (829 and 486 ng/mL), malignancy under treatment (293 ng/mL), hemodialysis within 1 year (35 ng/mL), peritoneal dialysis (55, 60 ng/mL), and suicide (312 ng/mL). Patients with malignancies who achieved complete cure at baseline were included in the study. The final analysis included 44 patients.

**Figure 2:**
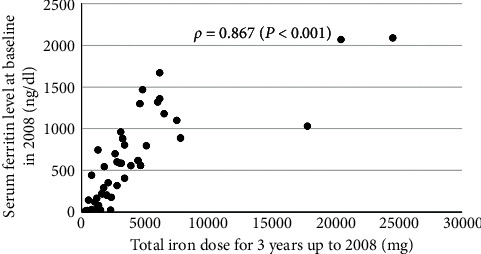
Relation between total iron dose injected in three years and serum ferritin level. *ρ*, Spearman's rank correlation coefficient.

To investigate the relationship of serum ferritin levels with total dosage of iron, we calculated the total amount of cideferron injected intravenously during the three-year period from April 2005 to March 2008 for all patients. The total amount of iron administered during the three years ranged from 300 to 24550 mg (8–682 mg/month), and there was a strong positive correlation between serum ferritin level and total iron dose by Spearman's rank correlation coefficient analysis (*n* = 44, *ρ* = 0.867, *p* < 0.001) (see Figure 2). Therefore, these patients were diagnosed with iatrogenic hyperferritinemia due to intravenous iron therapy. Considering that a subset of clinicians did not follow the 2004 JSDT guidelines for renal anemia [10], approximately half of our patients had high ferritin levels due to intravenous iron.

The average life expectancy of dialysis patients in industrialized countries is four years [4], whereas that of Japanese hemodialysis patients is much longer at around 10 years for patients in their 60s [12]; therefore, the follow-up period was 10 years in the current study.

The 2008 JSDT guidelines recommend iron administration in patients with a transferrin saturation (TSAT) level of ≤20% and a serum ferritin level of ≤100 ng/mL [13]. The recommended frequency of administration was up to once a week for three months or a total of 13 times at every dialysis session with the consideration of hemoglobin levels [13]. Therefore, we examined hemoglobin, TSAT, and ferritin every month and used 50 mg of cideferron (approximately in August 2011) or 40 mg of saccharated ferric oxide (approximately in September in 2011) intravenously at the end of the dialysis session only in patients with a TSAT of ≤20% and a serum ferritin level of ≤100 ng/mL between April 2008 and March 2018.

We used two rHuEPO, epoetin-beta or darbepoetin-alfa. Total rHuEPO dose was calculated as 1 *μ*g darbepoetin-alfa corresponding to 200 IU epoetin-beta. None of the patients received regular blood transfusions.

We used four phosphate binders: calcium carbonate, sevelamer, lanthanum (2009∼), and ferric citrate hydrate (2014∼). We controlled serum albumin-corrected calcium (8.4–10.0 mg/dL), phosphorus (3.5–6.0 mg/dL), and intact parathyroid hormone concentration levels (60–240 pg/mL) by using alfacalcidol, maxacalcitol, cinacalcet, and etelcalcetide hydrochloride (2017∼) in 10 years. We used ferric citrate hydrate (2014∼) carefully so that ferritin would not exceed 300 ng/mL.

### 2.3. Clinical Data and Laboratory Parameters

Serum ferritin levels were measured prior to the first dialysis of the week performed on Monday or Tuesday once monthly, with the patient in a supine position. The baseline data from April 2008 were included in the study (see Table 1).

### 2.4. Endpoints

Primary endpoint was all-cause death. Secondary endpoints were rates of cerebrovascular and cardiovascular diseases (CCVDs) including myocardial infarction, stroke, and peripheral artery disease; severe infections requiring hospitalization; and new-onset malignancies requiring hospitalization.

### 2.5. Statistical Analysis

Data were presented as means ± standard deviation or medians with interquartile range. A *p* value of <0.05 was considered to indicate a statistically significant difference. For comparison of survival, cumulative survival probability from the study entry until the terminal event was estimated by the Kaplan–Meier method. The Cox proportional hazards model was used to assess relative risks for all-cause death and the onset of CCVDs. All analyses were conducted using SPSS version 22.0 for Windows (IBM Japan, Tokyo, Japan).

## 3. Results

### 3.1. Baseline Characteristics of the Study Cohort

Table 1 shows the baseline characteristics of all patients grouped according to serum ferritin levels. The hemodialysis vintage was significantly longer in those with higher ferritin levels than in those with lower ferritin levels (*p*=0.05). Body mass index was significantly lower in those with higher ferritin levels than in those with lower ferritin levels (*p*=0.02). The percentage of patients with a history of cardiovascular disease was significantly higher in patients with initial ferritin levels ≥1000 ng/mL than in those with initial ferritin levels <300 ng/mL (*p*=0.01). nPCR was significantly higher in those with higher ferritin levels than in those with lower ferritin levels (*p*=0.005). Additionally, Kt/V (*p*=0.02), blood flow rate (*p*=0.01), Fe (*p*=0.02), TSAT (*p*=0.001), and serum calcium level (*p*=0.03) were significantly higher in patients with initial ferritin levels ≥1000 ng/mL than in those with initial ferritin levels <300 ng/mL. The percentage of patients receiving rHuEPO at the baseline was significantly higher in those with higher ferritin levels than in those with lower ferritin levels (*p*=0.03), although there was no difference in baseline hemoglobin levels and rHuEPO dose per week between these groups.

### 3.2. Grouping by Ferritin Levels

The patients were categorized into groups based on baseline ferritin levels determined in April 2008. A previous study analyzed iron overload in long-term hemodialysis patients by defining a serum ferritin level of >1000 ng/mL as an indicator of iron overload [14]. We used the cutoff value of 1000 ng/mL as the highest value. The 2015 JSDT guidelines recommend iron administration that targets a serum ferritin level of <300 ng/mL in hemodialysis patients [15], whereas the 2012 Kidney Disease Improving Global Outcome guidelines have set the upper ferritin limit as 500 ng/mL for hemodialysis patients (evidence level, 2 C) [16]. Therefore, we used the cutoff value of <300 ng/mL as a safe value and >500 ng/mL as a high value.

**Figure 3:**
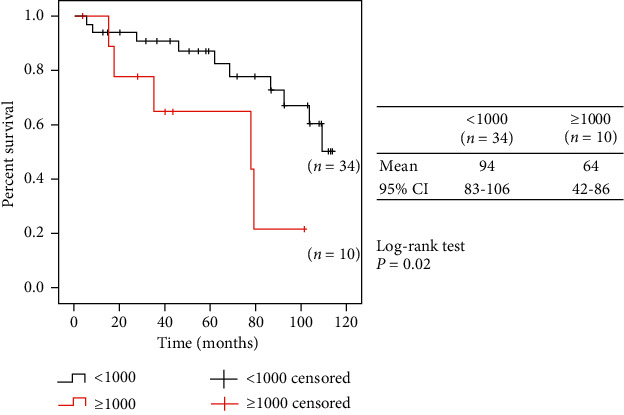
Kaplan–Meier survival curves for cerebrovascular and cardiovascular diseases according to serum ferritin level at baseline.

We compared two groups on the basis of ferritin levels: <1000 ng/ml (*n* = 34) vs. ≥1000 ng/ml (*n* = 10); the groups were compared regarding the risk of death, CCVDs, infection, and malignancy. Only the risk of CCVDs was significantly higher in patients with initial serum ferritin levels ≥1000 ng/mL than in the remaining patients (see Figure 3).

We observed the change in ferritin levels in the high-ferritin group (>500 ng/mL in 2008, *n* = 25) for 10 years. There were two subgroups under the high-ferritin group: one in which ferritin levels decreased to <300 ng/ml (*n* = 21) and the other in which ferritin levels did not decrease to <300 ng/ml (*n* = 4) during 10 years. We compared the prognosis of the two subgroups. In addition, we observed the group (300 ng/mL ≤ initial ferritin < 500).

### 3.3. Prognosis of the Entire Cohort

At the end of 10-year observation period, 24 patients died and 20 patients were alive. There were 16, 18, and 12 patients with CCVDs, severe infections, and new-onset malignancies, respectively (see Figure 1). The minimum follow-up duration was four months. First, we investigated whether patients with initial serum ferritin levels ≥1000 ng/mL had a significantly higher risk of death, CCVDs, severe infection, or new-onset malignancy compared with the remaining patients.

As shown in Figure 3, the risk of CCVDs was significantly higher in patients with initial serum ferritin levels ≥1000 ng/mL compared with the remaining patients. The history of cardiovascular disease at baseline (see Table 1) was more frequent in patients with initial ferritin levels ≥1000 ng/mL than in those with initial ferritin <300 ng/mL. This was associated with the fact that the history of end-stage renal disease (hemodialysis vintage) of the patients with initial ferritin levels ≥1000 ng/mL was longer than in those with initial ferritin <300 ng/mL. In addition, they were also more susceptible to CCVDs over the 10-year follow-up period.

Although there was no significant increase in the risk of new-onset malignancies, many patients with hyperferritinemia were diagnosed with various malignancies including ovarian cancer, gastrinoma, thyroid cancer, lung cancer, colon cancer, gastric cancer, and renal cell carcinoma.

### 3.4. Changes in Ferritin Levels of Patients with Initial Serum Ferritin >500 ng/mL during the 10-Year Follow-Up Period

**Figure 4:**
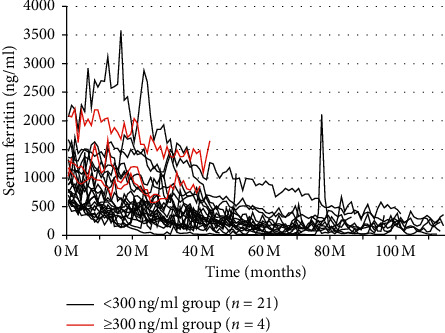
Changes in serum ferritin levels over 10 years of follow-up in patients with serum ferritin levels >500 ng/mL at baseline. Red lines: the serum ferritin levels did not decrease to <300 ng/mL during the 10-year follow-up period.

As shown in Figure 4, during the 10 years of follow-up, the ferritin levels fluctuated and slowly declined to <300 ng/mL in a median of 24.2 (10.5–46.5) months without any treatment in nearly all patients. However, regardless of the initial values, some patients whose initial ferritin values did not decrease easily were hospitalized several times either due to new CCVDs, infections, or new onset of malignancy. Especially, 4 of the 10 patients with initial ferritin levels >1000 ng/mL, and whose serum ferritin did not decrease easily, had a poor prognosis.

As shown in Table 2, the median ferritin levels were 797 (591–1210) ng/mL in the group that achieved a reduction of ferritin to <300 ng/mL and 1240 (1068–1893) ng/mL in the group that did not achieve the same reduction.

### 3.5. Risk of Death in Patients with Hyperferritinemia

Table 3 shows the Cox proportional hazards model to assess the risk of death in these two groups after correcting for age, which revealed that the risk of all-cause death was significantly higher in patients whose ferritin levels did not decrease to <300 ng/mL compared to those whose ferritin levels decreased to <300 ng/mL (hazard ratio, 9.6). In our additional analysis about the group (300 ng/mL ≤ initial ferritin < 500) too, HR was high.

## 4. Discussion

In the initial sample-size estimation for the study, we assumed 10-year rates of 40%, 30%, and 25% for CCVDs, severe infections, and new-onset malignancies, respectively. Further, 10 patients per variable were necessary for logistic regression analysis. Based on the predicted 10-year rate of 25% for the outcome of new-onset malignancies, a minimum of 40 patients were required for the cohort; therefore, the cohort size of the study was sufficient.

In the present study, we found that the risk of CCVDs was higher in patients with initial ferritin levels ≥1000 ng/mL than in those with initial ferritin levels <1000 ng/mL. We also determined that the majority of patients with high ferritin levels recovered without any treatment. However, further analysis revealed that the risk of all-cause death rate was significantly higher in four patients whose serum ferritins did not decrease to <300 ng/mL over the 10-year period compared with the remaining patients whose ferritin levels decreased to <300 ng/mL.

In the baseline data, the hemodialysis vintage was significantly longer in those with higher ferritin levels than in those with lower ferritin levels because they were administered more iron during their longer dialysis histories. High nPCR, Kt/V, and blood flow rate are characteristics of well-controlled long-term dialysis patients. Their higher serum calcium levels were due to taking more calcium carbonate, but their serum Ca levels were lower than 10.0 mg/dL, within the target level. In 10 years, almost all the patients achieved these target values.

Patients with initial ferritin levels ≥1000 ng/mL repeated hospitalization due to recurrent CCVDs or infections or new malignancy. For example, a 58-year-old female patient repeated cerebral hemorrhage and died of the third cerebral hemorrhage though she did not have hypertension. A 61-year-old female patient without diabetes repeated severe infection and died of septic shock.

Previous studies reported that the rate of CCVDs was higher in hemodialysis patients with high ferritin levels compared to those with low ferritin levels [17–19]. Elevated levels of hepcidin-25, which can activate macrophages, have been linked to cardiovascular events in dialysis patients, indicating that an increase in hepcidin-25 might be a mediator of cardiovascular morbidity in dialysis patients with iatrogenic iron overload [4]. Additionally, oxidative stress and arterial and cardiac structural changes might act synergistically to increase mortality and CCVDs in hemodialysis patients with iron overload. Oxidative stress due to intravenous iron infusion and iron overload might represent a “second hit” on the vascular bed [20]. Furthermore, iron-induced calcification in vascular smooth muscle cells via interleukin 24 has been reported to be increased during iron-induced calcification [21]. As shown in Table 1, the past history of CCVDs at baseline was more frequent in patients with initial ferritin levels ≥1000 ng/mL than in those with <300 ng/mL. These patients with initial ferritin levels ≥1000 ng/mL had much longer dialysis vintage than the other patients and were already administered more total intravenous iron for longer years before 2008 because all the patients were injected iron just after the start of hemodialysis. The patients in our study did not receive intravenous iron supplementation until their serum ferritin levels reduced to below 100 ng/mL for 10 years according to Japanese guidelines after 2008. We could not exclude the effects of dialysis vintage and past CCVDs before initiating the study; however, in Figure 3, the risk of CCVDs was significantly higher in these patients with initial serum ferritin levels ≥1000 ng/mL compared with the other patients during the follow-up period, suggesting the persistent damage to the vascular bed over several years by hyperferritinemia due to iron.

We also found that the elevated ferritin levels declined in majority of the patients without any treatment. The observation period for the natural course of iatrogenic hyperferritinemia in the present study is much longer than those reported previously [19, 20]. It is noteworthy that the hemoglobin levels were maintained within target values (hematocrit, 30%–33%) without any intravenous iron supplements throughout the 10 years of observation, suggesting that high ferritin levels in maintenance hemodialysis patients are reversible and that tissue iron might be utilized together with rHuEPO for hemoglobin synthesis. Only three patients with initial ferritin levels ≥1000 ng/mL could stop rHuEPO temporarily for 1–3 months during 10 years. However, groups with hyperferritinemia could not save the average total amount of rHuEPO used during the next 10 years.

We also found that the risk of all-cause death rate was significantly higher in the patients whose serum ferritin levels did not decrease to <300 ng/mL compared with the remaining patients whose ferritin levels decreased to <300 ng/mL. In a cohort of 90 Japanese hemodialysis patients, Hasuike et al. found that a serum ferritin level of >100 ng/mL was associated with a higher mortality risk compared with a serum ferritin level of ≤100 ng/mL [9]. In a prospective cohort study including 1086 Japanese patients undergoing maintenance hemodialysis, Kuragano et al. found that the risk of death and/or adverse events was higher in patients with consistently high serum ferritin levels and in those with high-amplitude ferritin fluctuations [17]. Additionally, Kim et al. reported that a major rise in serum ferritin levels in patients with a baseline ferritin level ≥200 ng/mL and even a slight rise of serum ferritin in those with a baseline ferritin level ≥800 ng/mL during the first six months after hemodialysis initiation were associated with higher mortality [22]. Furthermore, Son et al. showed that a ferritin level >100 ng/mL was associated with increased rates of all-cause mortality and cardiovascular events [19]. In addition to these findings from previous studies, we found that extremely high ferritin levels due to excessive iron therapy were associated with an increased risk of all-cause death especially in patients whose ferritin levels did not decline.

The JSDT has proposed that dialysis patients should receive a minimal amount of intravenous iron and only in the presence of true iron deficiency and has warned against maintenance intravenous iron therapy due to toxicity concerns [23]. Discrepancies and controversy remain between Japan and other countries regarding optimal ferritin levels. Possible explanations of these conflicting findings include differences in methods of adverse event adjudication, baseline differences in patient characteristics, and short follow-up periods in the majority of the studies [11].

In the present study, hyperferritinemia was induced by excessive iatrogenic administration of intravenous iron. Our patients in the high-ferritin group (>500 ng/mL) were administered >2900 mg cumulative iron dose during the 3 years (>81 mg/month). The monthly dose of iron (>81 mg/month) in our high-ferritin group is much less than the low-dose group in the PIVOTAL study [6] (145 mg/month interquartile range, 100–190). The present study findings confirm that a minimal amount of intravenous iron should be administered to avoid hyperferritinemia, even if the condition is transient. Despite the limited small sample size, this study is very important. More large-scale and long-term observational studies are needed to confirm our findings about the risk of hyperferritinemia.

The present study has several limitations. First, the study included a small cohort and was conducted at a single center. Second, the current study results are not conclusive because of the low number of patients with very high serum ferritin levels due to excessive iron supplementation. Third, transient inflammation (*n* = 18, see Figure 1) or new-onset malignancy (*n* = 12, see Figure 1) during the 10 years of follow-up might have affected the risk of death or CCVDs although patients with chronic inflammation, malnutrition, or malignancies under treatment at baseline were excluded from the study.

## 5. Conclusions

The risk of CCVDs was higher in patients with intravenous iron therapy-induced hyperferritinemia despite the reduction in ferritin. Patients with intravenous iron therapy-induced hyperferritinemia and those with serum ferritin levels that did not decrease to <300 ng/mL were at a higher risk of all-cause death compared with the other patients.

## Figures and Tables

**Table 1:** Baseline data of all patients based on serum ferritin levels in 2008.

	All the patients (*n* = 44)	Ferritin <300 ng/mL (*n* = 15)	300 ng/mL ≤ ferritin < 1000 ng/mL (*n* = 19)	Ferritin ≥1000 ng/mL (*n* = 10)	*p* value for all
Serum ferritin					
Female	21 (48%)	8 (53%)	7 (37%)	6 (60%)	
Male	23 (52%)	7 (47%)	12 (63%)	4 (40%)	
Serum ferritin (ng/ml)	572 (184, 942)	122 (24, 203)	596 (554, 797)	1340 (1160, 1770)	—
Demographic characteristics					
Age (years)	64 ± 14	64 ± 16	66 ± 15	61 ± 11	0.7
Dialysis vintage (years)	4 (2, 11)	1 (1, 10)	3 (2, 17)	8 (4, 19)	0.05^∗^
BMI (kg/m^2^)	21 ± 4	23 ± 5	20 ± 2	19 ± 3	0.02^∗^
Systolic blood pressure (mmHg)	139 ± 16	143 ± 13	134 ± 17	144 ± 16	0.2
Complications at the baseline (*n*, %)					
Diabetes mellitus	18 (41%)	8 (53%)	7 (37%)	3 (30%)	0.5
Cardiovascular disease	10 (23%)	0 (0%)^†^	6 (32%)	4 (40%)^†^	0.01^∗^
Secondaryhyperparathyroidism	18 (41%)	6 (40%)	6 (32%)	6 (60%)	0.4
Dialysis amyloidosis	3 (7%)	0 (0%)	1 (5%)	2 (20%)	0.2
HCV or HB	6 (14%)	3 (20%)	2 (11%)	1 (10%)	0.7
Dialysis conditions					
Kt/V	1.5 ± 0.3	1.4 ± 0.3^‡^	1.5 ± 0.3	1.7 ± 0.3^‡^	0.02^∗^
nPCR (g/kg/day)	0.9 ± 0.1	0.8 ± 0.2^§^	0.9 ± 0.1^¶^	1.0 ± 0.1^§¶^	0.005^∗^
Membrane area of dialyzer (m^2^)	1.7 ± 0.3	1.6 ± 0.3	1.7 ± 0.3	1.8 ± 0.2	0.4
Blood flow rate (ml/min)	206 ± 23	194 ± 22††	208 ± 20	221 ± 22^††^	0.01^∗^
Dialysis time (hours)	3.8 ± 0.4	3.7 ± 0.4	3.8 ± 0.4	3.9 ± 0.3	0.3
Laboratory data					
Hb (g/dl)	11.1 ± 1.3	11.4 ± 1.8	10.8 ± 0.9	11.3 ± 1.0	0.4
Fe (*μ*mol/l)	73 (48, 114)	57 (45, 75)^‡‡^	73.0 (51, 155)	112.5 (81, 133)^‡‡^	0.02^∗^
TSAT (%)	36 (24, 57)	25 (16, 35)^§§^	36 (24, 63)	57 (45, 77)^§§^	0.001^∗^
Cr (mg/dl)	9.8 ± 2.6	9.1 ± 2.9	10.1 ± 2.4	10.2 ± 2.7	0.5
BUN (mg/dl)	64 ± 12	63 ± 13	62 ± 10	72 ± 11	0.07
Ca (mg/dl)	8.9 ± 0.7	8.6 ± 0.7^¶¶^	8.8 ± 0.6	9.4 ± 0.6^¶¶^	0.03^∗^
IP (mg/dl)	5.0 ± 0.9	5.1 ± 0.7	5.1 ± 1.0	4.7 ± 1.0	0.4
K (mEq/L)	4.6 ± 0.6	4.6 ± 0.6	4.5 ± 0.6	4.8 ± 0.6	0.6
Alb (g/dl)	3.7 ± 0.3	3.6 ± 0.3	3.7 ± 0.2	3.8 ± 0.2	0.09
T.chol (mg/dl)	161 ± 30	168 ± 26	161 ± 36	151 ± 24	0.4
CRP (mg/dl)	0.1 (0.0, 0.3)	0.1 (0.0, 0.3)	0.1 (0.0, 0.4)	0.1 (0.1, 0.2)	0.9
*β*2MG (mg/L)	27 ± 7	26 ± 8	27 ± 6	30 ± 6	0.4
Intact PTH (pg/ml)	112 (59, 214)	165 (75, 230)	110 (58, 210)	90 (48, 170)	0.5
Medication at start (*n*, %)					
ACEI and/or ARB	19 (43%)	5 (33%)	8 (42%)	6 (60%)	0.4
Intravenous iron administration	7 (16%)	3 (20%)	4 (21%)	0 (0%)	0.4
rHuEPO	38 (86%)	10 (67%)	18 (95%)	10 (100%)	0.03^∗^
Dose, IU/week at baseline	3341 ± 2402	2950 ± 2813	3513 ± 2476	3600 ± 1613	0.7
Total dose (IU) in 10 years	1676182 ± 1151833	1465167 ± 1029122	1935961 ± 1242785	1499125 ± 1162216	0.4

BMI, body mass index; HCV, hepatitis C virus; HB, hepatitis B virus; Kt/V, clearance × time/volume; nPCR, normalized protein catabolism rate; Hb, hemoglobin; TSAT, transferrin saturation; Cr, creatinine; BUN, blood urea nitrogen; Ca, calcium; IP, inorganic phosphorus; K, potassium; Alb, albumin; T.chol, total cholesterol; CRP, C-reactive protein, *β*2MG, *β*2-macroglobulin, PTH, parathyroid hormone; ACEI, angiotensin-converting enzyme inhibitor; ARB, angiotensin II receptor blocker; rHuEPO, recombinant human erythropoietin. Data are presented as *n*(%), mean ± standard deviation, and median (interquartile range); multiplicity of comparisons among groups was accounted by Bonferroni correction. ^*∗*^*p* < 0.05. †, ‡, §, ¶, ††, ‡‡, §§, and ¶¶: significant differences observed by *n* comparison between two groups.

**Table 2:** Comparison of patients with initial ferritin levels >500 ng/mL categorized by ferritin levels at the end of the 10-year follow-up period.

	*n*		<300 ng/mL	*n*		≥300 ng/mL	*p* value
Sex	21			4			0.6^a^
Male		11	(52%)		1	(25%)	
Female		10	(48%)		3	(75%)	
Primary disease	21			4			0.8^a^
ADPKD		0	(0%)		0	(0%)	
AKI		0	(0%)		0	(0%)	
DM		8	(38%)		1	(25%)	
Glomerulonephritis		9	(43%)		3	(75%)	
IgA nephropathy		3	(14%)		0	(0%)	
Renal tuberculosis		1	(5%)		0	(0%)	
Nephrosclerosis		0	(0%)		0	(0%)	
Mortality (1000 person-year)	21	71		4	205		–
Incidence of infection (1000 person-year)	21	37		4	110		–
Incidence of CCVD (1000 person-year)	21	89		4	134		–
Incidence of malignancy (1000 person-year)	21	65		4	0		–
Age	21	63	(54, 73)	4	60	(48, 72)	0.5^b^
Dialysis vintage	21	6	(3, 18)	4	8	(3, 22)	0.9^b^
Initial ferritin (ng/ml)	21	797	(591, 1210)^∗^	4	1240	(1068, 1893)^∗^	0.05^b^
Months to decrease below 300 ng/ml	21	24	(11, 47)^∗^		4	–	–^b^

ADPKD, autosomal dominant polycystic kidney disease; AKI, acute kidney injury; DM, diabetes mellitus; CCVD, cerebrovascular and cardiovascular diseases. Data are presented as *n* (%), mean ± standard deviation, and median (interquartile range). *p* values: a, Fisher's exact test; *b*, the Mann–Whitney *U* test. ^*∗*^*p* < 0.05.

**Table 3:** Cox proportional hazards model for risk of death based on ferritin level <300 ng/mL versus ≥300 ng/mL.

	Univariate analysis					Corrected by age				
	HR	95% CI			*p* value	HR	95% CI			*p* value
Sex female (vs. male)	0.8	0.3	,	2.0	0.6	−				
Age	1.1	1.0	,	1.1	**0.01** ^∗^	−				
Dialysis vintage	1.0	1.0	,	1.1	0.3	−				
Initial ferritin ≥500 at start										
Decreased to <300 ng/mL in 10 years	1.0		Ref			1.0		Ref		
Not decreased: ≥300 ng/mL in 10 years	7.6	1.2	,	47.7	**0.03** ^∗^	**9.6**	1.4	,	65.1	**0.02** ^∗^
300 ng/mL ≤ initial ferritin < 500 at start										
Decreased to <300 ng/mL in 10 years	1.0		Ref			1.0		Ref		
Not decreased: ≥300 ng/mL in 10 years	5.2	1.1	,	25.8	**0.04** ^∗^	11.6	2.0	,	67.1	**0.006** ^∗^

HR, hazard ratio; CI, confidence interval. ^*∗*^*p* < 0.05.

## Data Availability

The data used to support the findings of this study are available from the corresponding author upon request.
